# A Detailed Systematic Review Comparing Patent Foramen Ovale Closure vs Medical Therapy for the Prevention of Recurrent Cryptogenic Stroke

**DOI:** 10.7759/cureus.65632

**Published:** 2024-07-29

**Authors:** Amna A Mirza, Moyal Z Saad, Parikshit Bittla, Sai Pavitra Paidimarri, Shriya Ayuthu, Yashkumar D Chauhan, Safeera Khan

**Affiliations:** 1 Medicine, Ziauddin Medical College, Karachi, PAK; 2 Internal Medicine, Jinnah Medical and Dental College, Karachi, PAK; 3 Internal Medicine, Bhaskar Medical College, Hyderabad, IND; 4 Internal Medicine, Tianjin Medical University, Tianjin, CHN; 5 Medicine, Kamineni Institute of Medical Sciences, Nalgonda, IND; 6 Medicine, Smt. Nathiba Hargovandas Lakhmichand (NHL) Municipal Medical College, Ahmedabad, IND; 7 Internal Medicine, California Institute of Behavioral Neurosciences and Psychology (CIBNP), Fairfield, USA

**Keywords:** antiplatelets, stroke, surgical treatment, anticoagulants, patent foramen ovale (pfo)

## Abstract

Cryptogenic stroke refers to a type of ischemic stroke with no identifiable cause despite extensive diagnostic testing. Patent foramen ovale (PFO) treatment modality for the prevention of cryptogenic stroke has been controversial. We undertook this systematic review to compare the efficacy of PFO closure versus medical therapy in preventing recurrent cryptogenic stroke and to provide insight into the most effective treatment modality. Inclusion criteria included patients who had PFO, papers written in English language or had translation available, and papers focusing on medical therapy including drug and surgical treatment for PFO for the prevention of recurrent stroke. Exclusion criteria included articles in which full text could not be obtained and articles in which only one treatment modality was mentioned, either surgical closure or drug therapy. The databases used were PubMed, Cochrane, Embase, and ClinicalTrials.gov. We conducted a bias assessment through the modified Jadad scale for randomized controlled trials (RCTs) and AMSTAR.Ca for meta-analysis and systematic review. The literature search identified a total of 277 papers. After screening, 12 papers were selected for the review. Among these, five were RCTs, five were meta-analyses, one was a systematic review, and one was a systematic review with network meta-analysis. The RCTs included a total of 3,336 participants, while the meta-analyses included 21,813 participants. These finalized papers examined the outcomes of PFO closure compared to medical therapy in preventing recurrent strokes.

## Introduction and background

Stroke occurs when the blood supply to the brain is altered by either a clot or rupture, reducing the supply of blood to the affected part. Stroke can be ischemic when a thrombus or embolus stops the flow of blood or hemorrhagic when the blood vessel wall ruptures. Stroke is the second leading cause of death worldwide and the fifth leading cause of death in the USA [1-3]. Several risk factors contribute to stroke, including hypertension, diabetes mellitus, myocardial ischemia, atrial fibrillation, smoking, physical inactivity, and increased blood lipids [4,5]. Sometimes, there are no identifiable causes of stroke, which is then referred to as cryptogenic stroke. Patent foramen ovale (PFO) has been linked as a cause of cryptogenic stroke when no other cause can be found [1]. Nearly 50% of cryptogenic strokes are associated with PFO [6].

PFO is normal before birth but may fail to close after birth, forming a connection between the right and left atria, causing increased blood flow to the right atria and lungs. Sometimes, due to pressure changes, blood can flow from the right atrium to the left atrium, carrying a clot that can lead to a stroke [1]. PFO closes due to the fusion of the septum primum and secundum after the pressure in the left atrium increases, preventing the shunting of blood from the right to the left side [7]. There is significant diversity in the rate of PFO in the population [1]. PFO has been identified as a risk factor for stroke in younger populations, and there has always been a debate regarding the best modality for the prevention of recurrent cryptogenic stroke: surgical closure, antiplatelet therapy, or anticoagulant therapy [8].

Treatment modalities like closure and medical therapy compare the best methods for preventing recurrent stroke in patients with a history of PFO. Several studies have explored the best modality for the treatment of PFO, either closure or medical therapy alone, with inconsistent findings [8,9]. In many patients, PFO closure has shown benefits in preventing episodes of recurrent stroke, while in some, anticoagulants and antiplatelets have shown benefits [10]. Some studies have shown the superiority of PFO closure compared to antiplatelets and anticoagulants, while other studies have shown no difference in outcomes or inconclusive evidence. Closure might also be associated with a higher risk of complications like atrial fibrillation, which is also a risk factor for embolic stroke, and even device-related complications have been reported [11-13].

To resolve the conflicting evidence and provide better care for physicians, we conducted this updated systematic review to determine better outcomes for patients with PFO and stroke, using either medical therapy or surgical closure to prevent the recurrence of cryptogenic stroke.

## Review

Methods

Search Strategies

This systematic review was conducted using the Preferred Reporting Items for Systematic Reviews and Meta-Analyses (PRISMA) 2020 guidelines. The authors searched PubMed, Cochrane, and ClinicalTrials.gov to search for relevant literature pertaining to the topic. Two different search strategies were employed in PubMed to ensure a comprehensive capture of relevant articles. PubMed Mesh strategy used Foramen Ovale, Patent/drug therapyMajr AND Foramen Ovale, Patent/surgery MajrANDStrokeMesh (((patent foramen ovale AND surgical closure)) AND (patent foramen ovale AND medical therapy)) AND (stroke). The search strategies for various databases are listed below (Table 1).

**Table 1:** Search strategies and database results

Keywords search strategy	Database used	Results
(((patent foramen ovale AND surgical closure)) AND (patent foramen ovale AND medical therapy)) AND (stroke)	PubMed	167
Foramen Ovale, Patent/drug therapyMajr AND Foramen Ovale, Patent/surgery MajrANDStrokeMesh	PubMed	92
PFO - Patent Foramen Ovale Stroke Medical treatment	ClinicalTrials.gov	9
Patent foramen ovale medical therapy vs drug therapy for stroke prevention	Cochrane	9

Inclusion Criteria

We included all the studies which recorded patient and aggregate-level data of individuals with PFO undergoing either surgical closure or medical intervention for subsequent stroke prevention. Randomized controlled trials (RCTs), cohorts, case-control studies, and meta-analyses/systematic reviews were reviewed. We selected patients who had PFO and a history of stroke. Papers written in the English language or had translations available in English were used. Papers that focused on medical therapy including drugs (antiplatelets and anticoagulation) and surgical treatment for PFO for the prevention of recurrent stroke were selected. All studies that were included had human participants.

Exclusion Criteria

Moreover, studies were excluded if they bore insufficient data on the clinical progression of the condition, had insufficient patient follow-up, or lacked a comparison group. All non-English or those without available translated versions were excluded. Articles in which full text could not be obtained were excluded. Articles in which both intervention surgical closure of PFO and drug therapy were not mentioned were excluded.

Selection Process

Duplicates were removed both through the end note and manually by the author. The authors reviewed the duplicate abstracts and titles before removing. Studies which included RCT, meta-analysis, and systematic reviews were included with full-text articles. The articles that included a comparison between the closure of PFO in comparison to medical therapy for the prevention of recurrent stroke were selected for review. All studies underwent full-text evaluation. Two authors screened the title, abstract, and full texts of studies in duplicate. Any conflict in the study selection was, thereafter, resolved by a senior author.

Results

**Figure 1:**
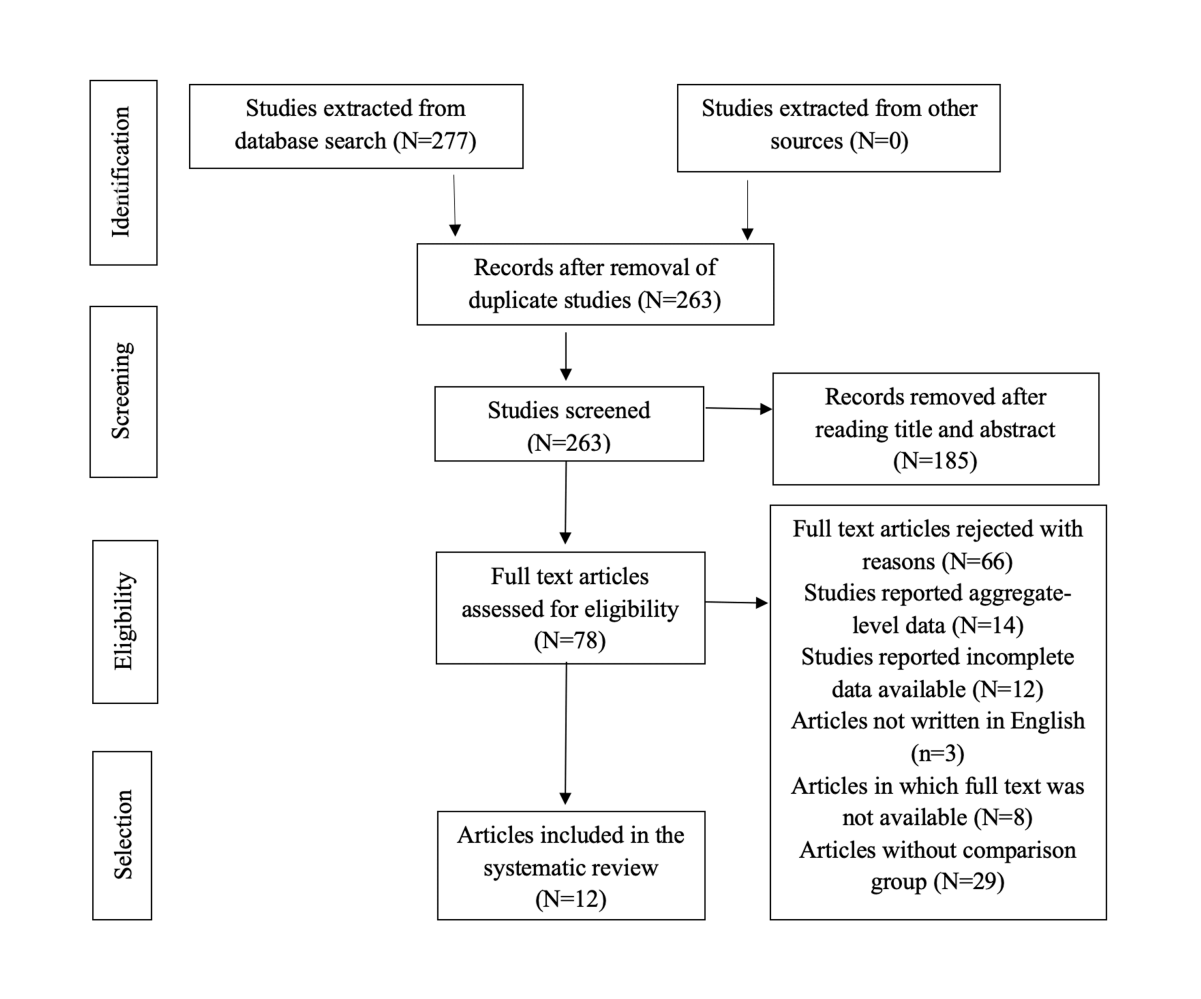
PRISMA flow diagram PRISMA: Preferred Reporting Items for Systematic Reviews and Meta-Analyses

A total of 277 articles were found across all databases. The literature search yielded PubMed (n=259), Cochrane (n=9), and ClinicalTrials.gov (n=9). No studies were extracted from other sources. After resolving disagreements regarding study selection, studies were entered into an Excel sheet, where 14 duplicate articles were removed. A total of 185 articles were rejected after reviewing their titles and abstracts. In the final phase of selection, full-text versions of 78 studies were read for clarity. The articles were screened for relevance to the topic, and 21 articles were shortlisted. Articles were excluded for reporting insufficient patient follow-up duration (n=14), incomplete patient data (n=12), or lack of a comparison group (n=29). The shortlisted articles were further evaluated for relevance through quality appraisal and availability of full texts, resulting in 12 articles identified for the review process. The selection process is shown through a PRISMA flow diagram (Figure 1).

Data Extraction

Selected studies were transferred to an Excel sheet, and a table was formed after the removal of duplicate studies. Extracted data came under the headings of author, study type, number of participants, participants per arm (closure/antiplatelets/anticoagulation), average age, average age per arm (closure/antiplatelets/anticoagulation), intervention, and conclusion. The selected studies varied in quality, and no set criteria for assessment or reporting of the condition was devised. Consequently, inconsistencies in their reporting methods were noted. The articles that were finalized for systematic review were reviewed for mean ages, number of patients, and therapeutic comparison between surgical closure and drug therapy.

Study Characteristics

A total of 12 papers were reviewed, encompassing 31,868 participants. Among these finalized studies, five were RCTs [11-15], one was a systematic review [6], five were meta-analyses, and one was a systematic review with network meta-analysis. All studies involved patients with PFO and a prior history of stroke. The selected studies focused on comparing the most effective modality for the prevention of recurrent stroke, through either surgical closure or medical therapy with antiplatelets and anticoagulants (Table 2) [11-22].

**Table 2:** Characteristics of included studies comparing PFO closure vs medical therapy for the prevention of recurrent cryptogenic stroke PFO: patent foramen ovale; RCT: randomized controlled trial; meta-analysis: a statistical analysis that combines the results of multiple scientific studies; N/A: not available

Article number	Research papers	Attributes	Type of study	Comparison in study	Number of participants, participants per arm (closure/antiplatelets/anticoagulation)	Average age, average age per arm (closure/antiplatelets/ anticoagulation)	Conclusion
1	Saver et al. 2017 [11]	Patients with prior PFO assigned to either closure or antiplatelets and anticoagulants	RCT	Comparing outcomes of drug therapy vs closure of PFO	980, closure: 499/antiplatelets: 481	45.9, closure: 45.9/antiplatelets: 45.8	PFO closure was associated with a lower rate of stroke than with medical therapy alone
2	Sondergaard et al. 2017 [12]	PFO closure with antiplatelet therapy in patients with prior stroke compared to antiplatelet therapy only	RCT	PFO closure or antiplatelet therapy for cryptogenic stroke	664, closure: 441/antiplatelets: 223	45.2, closure: 45.2/antiplatelets: 44.8	Closure of PFO yielded better outcomes than medical therapy alone
3	Furlan et al. 2012 [13]	PFO closure with antiplatelet therapy vs anticoagulation vs antiplatelets	RCT	Closure or medical therapy for cryptogenic stroke with PFO	909, closure: 312/antiplatelets: 309/ anticoagulation: 288	Closure: 46.0/antiplatelets: 45.8/anticoagulation: 46.2	No difference was noted with the closure of PFO plus antiplatelet therapy or medical therapy alone
4	Lee et al. 2018 [14]	PFO closure compared to medications	RCT	Device closure vs medical therapy	120	51.8	Closure resulted in a lower risk of stroke
5	Mas et al. 2017 [15]	Antiplatelet closure compared to anticoagulants	RCT	PFO closure or anticoagulation vs antiplatelets after stroke	663, closure: 238/antiplatelets: 235/anticoagulation: 190	Closure: 42.9/antiplatelets: 43.8/anticoagulation: 44.7	PFO and medical therapy with antiplatelets are associated with a lower risk of recurrent stroke
6	Spencer et al. 2014 [16]	PFO closure or antiplatelets or anticoagulant therapy	Systematic review	Cryptogenic stroke in patients with PFO	2303	45.4	Closure is not associated with a greater reduction of risk for neurological events
7	Udell et al. 2014 [17]	PFO closure and antithrombotic therapy	Meta-analysis	PFO closure vs medical therapy and heterogeneity in the results	2303	45.7	Transcatheter closure does not reduce the risk of cryptogenic stroke
8	Kheiri et al. 2019 [18]	PFO closure compared with antiplatelets or anticoagulants	Meta-analysis	Comparing PFO closure with medical therapy for the secondary prevention of cryptogenic stroke	3440	45.2	PFO closure is associated with a lower risk of recurrent stroke but is associated with greater side effects like atrial fibrillation
9	Mir et al. 2018 [19]	PFO closure vs anticoagulants vs antiplatelets	Systematic review and network meta-analysis	Comparing PFO closure with medical therapy for the secondary prevention of cryptogenic stroke	4416	N/A	PFO closure reduces the risk of stroke with antiplatelet therapy alone but may not show any difference with outcomes when compared to anticoagulation
10	Agarwal et al. 2012 [20]	Comparison between PFO closure and medical therapy with antiplatelet and anticoagulants	Meta-analysis	Closure of PFO via transcatheter compared to medical treatment	10327	N/A	More trials are needed to compare what treatment would be better
11	Ahmad et al. 2018 [21]	PFO closure compared to medical therapy	Meta-analysis	PFO closure compared to medical therapy	3440	45.3±9.7	The risk of recurrent stroke is lesser with PFO closure
12	Riaz et al. 2013 [22]	Transcatheter PFO closure versus medical therapy for cryptogenic stroke	Meta-analysis	PFO closure vs medical therapy	2303	N/A	PFO closure vs medical therapy showed more benefits

Statistical Analysis

Our study provided comprehensive data on patients with PFO who underwent surgical closure or medical therapy for the prevention of stroke recurrence. This included information on study type, number of patients, mean age, intervention, and conclusion (Table 2). Mean and standard deviation of age were calculated, while other variables were expressed as a percentage of the total number of their responses.

Quality Assessment of Study

Quality assessment was conducted using the AMSTAR tool for systematic reviews and meta-analyses and the modified Jadad scale for RCTs. The AMSTAR tool evaluates the methodological quality of systematic reviews and meta-analyses based on a series of 11 items, including the comprehensiveness of the literature search, the presence of a priori design, and the assessment of publication bias. Studies that met the criteria for quality appraisal were included in the systematic review, while those that did not meet the quality standards were excluded to ensure the robustness and reliability of the findings. For the RCTs, the modified Jadad scale was employed to assess the quality. This scale evaluates RCTs based on eight criteria, including randomization, blinding, and withdrawals/dropouts, among others. Each qualitative answer was converted into a numeric score (+1, 0, or -1), with higher scores indicating better methodological quality. Quality assessment was conducted independently by two reviewers to minimize bias and enhance the accuracy of the evaluation. In cases of disagreement, a third reviewer was consulted, and the final score was determined through consensus.

The modified Jadad scale had eight questions that were answered as either Yes, No, or Not Described. These questions covered essential aspects of the study design, such as whether the randomization process was described and appropriate, whether the study was double-blind, and whether there was an adequate description of dropouts and withdrawals. The included RCTs (n=5) had a mean score of 6.4±0.49, with individual scores ranging from 6 to 7, indicating generally high methodological quality among the included trials (Table 3) [11-15].

**Table 3:** Quality assessment of included studies using the modified Jadad scale

Article number	Research papers	Quality assessment score
1	Saver et al. 2017 [11]	7
2	Sondergaard et al. 2017 [12]	7
3	Furlan et al. 2012 [13]	6
4	Lee et al. 2018 [14]	6
5	Mas et al. 2017 [15]	6
6	Spencer et al. 2014 [16]	8
7	Udell et al. 2014 [17]	7
8	Kheiri et al. 2019 [18]	7
9	Mir et al. 2018 [19]	8
10	Agarwal et al. 2012 [20]	7
11	Ahmad et al. 2018 [21]	8
12	Riaz et al. 2013 [22]	7

Discussion

In the current systemic analysis, five RCTs, five meta-analyses, one systematic review, and one meta-analysis and systematic review combined with network meta-analysis were reviewed. Our findings indicated a relative benefit of PFO closure in preventing recurrent episodes of neurological events. However, it is important to note that some meta-analyses may include data from the RCTs we reviewed. The results of one or two large RCTs could influence the meta-analyses towards favorable conclusions for PFO closure. Therefore, the percentage of studies (60%) showing favorable results for closure may not be entirely accurate. In contrast, 33% of the studies found no significant difference in stroke prevention between anticoagulant or antiplatelet therapy and PFO closure. This indicates that the evidence is not sufficient to conclusively determine the superiority of either treatment modality, highlighting the need for further evaluation and research [11-22].

Studies also reported an increase in the adverse event of atrial fibrillation/flutter; some reported no difference in adverse events like bleeding in both groups [17-19]. Some studies reported an increased risk of bleeding with anticoagulants as compared to some which reported no increased incidence in bleeding with medical therapy. No difference was reported in the risk of stroke compared to closure and anticoagulants [18,19]. Most of the studies reported the benefits of closure vs medical therapy alone. Closure increased the risk of atrial flutter and fibrillation. Previous meta-analysis emphasized the need for more studies to be conducted to discuss which treatment option would be better to prevent recurrent stroke [20]. Some studies have suggested that the successful outcome of PFO closure in preventing stroke is related to the size of PFO [14-21]. Subsequently, meta-analyses and clinical trials conducted have shown better outcomes with closure in preventing recurrent episodes of stroke as compared to medical therapy, but device closure is associated with a greater risk of side effects like atrial fibrillation or atrial flutter [17]. One randomized clinical trial reported a higher incidence of pulmonary embolism associated with closure, but the overall risk of stroke was lower compared to medical therapy. Some reported higher incidences of atrial fibrillation in the closure group compared to antiplatelet therapy but also reported a higher incidence of procedural complications, while some reported no difference in the primary endpoint.

Given the risks associated with antithrombotic therapy, particularly its potential to cause bleeding, it is essential to conduct further evaluation and exploration for patients with a higher risk of bleeding. This includes assessing the benefits of prescribing antiplatelets over anticoagulants for such patient groups. Further studies need to be done. Additionally, it was noted that the closure of PFO combined with the use of antiplatelets was associated with a lower risk of stroke. Furthermore, the lack of blinding in the open-label trials and inadequate follow-up and a higher dropout rate in the drug therapy group compared to the surgical group were significant limitations. If the events were more likely to occur in the groups that were lost to follow-up, the results will falsely favor the closure of PFO.

Some consideration was also drawn to the fact that there was unequal exposure of treatment in the assigned group, with more dropouts or failures to follow up in the drug therapy group compared to the closure group. Notably, data on each arm of the studies are crucial for accurately understanding the true impact of the treatment modalities. Considerations regarding the size of PFO, which varied in different studies, were also noted. Although 60% of studies reported better outcomes with PFO closure in preventing recurrent stroke, there are variations in the results, with some studies showing no difference in outcomes. This highlights the need for more studies to be conducted. Further evaluation is needed to determine the best treatment method and options, as outcomes may also be patient-dependent, such as larger PFOs being associated with a lower risk of stroke with PFO closure.

Limitations

Several limitations were identified in this systematic review. The shunt size varied across studies, with many not specifying it, making it difficult to assess its impact on treatment outcomes comprehensively. Additionally, there was variability in the devices used for PFO closure, which could influence the results and their applicability to clinical practice. The number of RCTs included was limited, reducing the robustness of the conclusions. Furthermore, the duration of follow-up differed among the studies, affecting the evaluation of long-term outcomes and the sustainability of treatment effects. These factors highlight the need for more standardized and high-quality research in this area.

## Conclusions

This systematic review compared PFO closure with medical therapy for the prevention of recurrent cryptogenic stroke. The findings suggest that PFO closure is associated with a lower risk of recurrent stroke in a majority of the studies reviewed. However, significant variability in outcomes exists, with some studies showing no difference between the two treatment modalities and others emphasizing the need for further evaluation. Additionally, device closure is associated with an increased risk of atrial fibrillation, which must be considered when choosing a treatment strategy. The review highlights the necessity for more comprehensive, larger-scale studies with longer follow-up periods to conclusively determine the most effective treatment modality. Factors such as the size of the PFO and individual patient risk profiles should also be considered. Overall, while PFO closure shows promise in reducing the risk of recurrent strokes, careful consideration of the associated risks and patient-specific factors is essential for optimizing treatment outcomes.
